# Evaluation of Electrochromic Properties of Polypyrrole/Poly(Methylene Blue) Layer Doped by Polysaccharides

**DOI:** 10.3390/s22010232

**Published:** 2021-12-29

**Authors:** Vilma Ratautaite, Raimonda Boguzaite, Migle Beatrice Mickeviciute, Lina Mikoliunaite, Urte Samukaite-Bubniene, Arunas Ramanavicius, Almira Ramanaviciene

**Affiliations:** 1 Laboratory of Nanotechnology, Department of Functional Materials and Electronics, Center for Physical Sciences and Technology, Sauletekio Ave. 3, LT-10257 Vilnius, Lithuania; vilma.ratautaite@ftmc.lt (V.R.); raimonda.boguzaite@ftmc.lt (R.B.); urte.samukaite-bubniene@chf.vu.lt (U.S.-B.); arunas.ramanavicius@chf.vu.lt (A.R.); 2 Department of Physical Chemistry, Faculty of Chemistry and Geosciences, Institute of Chemistry, Vilnius University, Naugarduko Str. 24, LT-03225 Vilnius, Lithuania; miglebeatricemick@gmail.com (M.B.M.); lina.mikoliunaite@chf.vu.lt (L.M.); 3 Laboratory of Spectroelectrochemistry, Department of Organic Chemistry, Center for Physical Sciences and Technology, Sauletekio Ave. 3, LT-10257 Vilnius, Lithuania; 4 NanoTechnas—Center of Nanotechnology and Materials Science, Faculty of Chemistry and Geosciences, Institute of Chemistry, Vilnius University, Naugarduko Str. 24, LT-03225 Vilnius, Lithuania

**Keywords:** methylene blue, polypyrrole (Ppy), polysaccharides, heparin, electrochromic polymers, caffeine, theophylline, theobromine

## Abstract

Polypyrrole (Ppy) and poly(methylene blue) (PMB) heterostructure (Ppy-PMB) was electrochemically formed on the indium tin oxide (ITO) coated glass slides, which served as working electrodes. For electropolymerization, a solution containing pyrrole, methylene blue, and a saccharide (lactose, sucrose, or heparin) that served as dopant was used. The aim of this study was to compare the effect of the saccharides (lactose, sucrose, and heparin) on the electrochromic properties of the Ppy-PMB layer. AFM and SEM have been used for the analysis of the surface dominant features of the Ppy-PMB layers. From these images, it was concluded that the saccharides used in this study have a moderate effect on the surface morphology. Electrochromic properties were analyzed with respect to the changes of absorbance of the layer at two wavelengths (668 nm and 750 nm) by changing the pH of the surrounding solution and the potential between +0.8 V and −0.8 V. It was demonstrated that the highest absorbance changes are characteristic for all layers in the acidic media. Meanwhile, the absorbance changes of the layers were decreased in the more alkaline media. It was determined that the Ppy-PMB layers with heparin as a dopant were more mechanically stable in comparison to the layers doped with lactose and sucrose. Therefore, the Ppy-PMB layer doped with heparin was selected for the further experiment and it was applied in the design of electrochromic sensors for the determination of three xanthine derivatives: caffeine, theobromine, and theophylline. A linear relationship of Δ*A* (∆*A* = *A*_+0.8V_ − *A*_−0.8V_) vs. concentration was determined for all three xanthine derivatives studied. The largest change in optical absorption was observed in the case of theophylline determination.

## 1. Introduction

Electrochromic properties are characteristic features not only for non-organic materials but also for some conjugated (conducting) polymers (e.g., polypyrrole (Ppy) and poly(methylene blue) (PMB) possess remarkable electrochromic properties). In conducting polymers, electrochromism occurs due to changes in the π-π conjugated electronic system and the ability to participate in electrochemical oxidation and reduction. Electrochromism describes the phenomenon in which the color of the material depends on the applied potential. The key parameters of the electrochromism are (i) response time; (ii) optical contrast; (iii) electrochromic efficiency; (iv) durability; and (v) optical memory [1].

Ppy is one of the most analyzed conducting polymers used for the development of various types of sensors. Ppy is easily synthesized by chemical [2,3,4], enzymatic [5,6], electrochemical [7,8,9], or other methods. Firstly, it was synthesized electrochemically by A. F. Diaz and K. Keiji Kanazawa. The resulting layer was characterized by appropriate adsorption and high conductivity [10]. 

**Figure 1:**
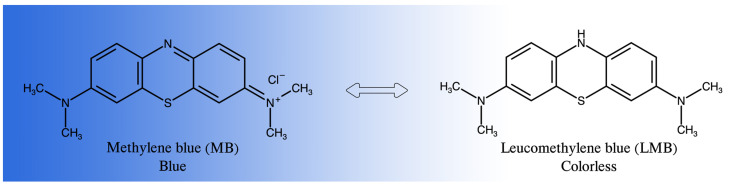
Chemical structures of methylene blue and leucomethylene blue.

Methylene blue (MB) is a phenothiazine (PT) derivative (Figure 1). This is a heterocyclic organic compound, which is well known for biomedical applications. In analytical chemistry, it is used as a redox mediator that undergoes color changes from bright blue to colorless when the oxidized form becomes reduced [11]. In previous studies, cyclic voltammetry was the mostly applied method for the electrochemical polymerization of PMB [11,12,13,14,15,16].

Overall, electrochemical polymerization has a remarkable advantage as polymer preparation method due to the ability to form layers with controllable properties on various types and shapes of electrodes. The controllable parameters of the electrochemical polymerization include the value of potential or current density, time, electrochemical method (cyclic voltammetry (CV), chronoamperometry, etc.). 

Moreover, the composition and pH of the polymerization mixture also have a significant influence on the properties, including electrochromic ones, of the polymer layer [17]. The conductivity of Ppy can be effectively tuned by a careful selection of dopants [18,19,20]. In most cases, small ions of strong acids are used as dopants, for instance, chloride [21], sulfate [8], perchlorate [22], iodine [23], and others. Small ions of a weak acid can be used as dopants too, for example, carboxylates [18]. Among the various dopants, saccharides are special substances [19,24,25]. The characteristics of polypyrrole-polysaccharide thin layers and the influence of three polysaccharides, namely, heparin, chondroitin-4-sulphate, and hyaluronic acid on the polypyrrole chemical and physicochemical properties were evaluated [19]. The polypyrrole layers in the aforementioned study were deposited on the ITO electrodes. This study found a strong correlation between the sample morphology and the current density during polymerization of Ppy in the presence of polysaccharides: a smooth surface morphology was observed when the current density was in the range of 100–700 μA/cm^2^, whereas high current (synthesis charge > 1.0 mA/cm^2^) or longer time (synthesis charge > 100 mC/cm^2^) led to form rough surfaces. It is noteworthy that in the above-mentioned study, extremely high concentrations of pyrrole (1 M) and doping anion (heparin, hyaluronic acid, or chondroitin-4-sulphate) (2.0 mg/mL) were used. Water, propylene carbonate, and acetonitrile were used as different solvents for the preparation of polymerization mixtures containing pyrrole and heparin as a polyanion [24]. The Ppy layer was deposited on the fluorine-doped tin oxide (FTO)-coated glass surface. The aforementioned study evaluated the electrochromic contrast and redox stability. Ppy layer doped by heparin was characterized by cyclic voltammetry and by in situ spectroelectrochemistry. It was concluded that the presence of heparin caused a drastic enhancement of electro-optical stability of Ppy. The evaluation of surface dominant features has demonstrated a Ppy and heparin layer composed of quasi-spherical grains (50–80 nm in dimensions). The layers were grown anodically at constant potential mode at 0.8 V vs. Ag/AgCl and by passing a charge of 100 mC from aqueous solution containing 0.1 M pyrrole and 0.01 mg/mL heparin. The previous works demonstrated the effect of polysaccharides on Ppy properties, but the understanding of the impact on the electric and electrochromic properties of Ppy can still be improved, for instance, stability and response speed.

Several studies describing the combination of Ppy and PMB have been published [26,27,28,29,30,31]. The results of electrochemical polymerization of pyrrole are better when the polymerization is carried out in an acidic solution, preferably ≤pH 7 [17,32]. The pH of the solution for polymerization of methylene blue is preferably around pH 6.0. An electrochemical activity of PMB after polymerization is well established as good and reversible in the pH region from 2.0 to 8.0 [14]. Usually, salts such as KCl, Na_2_SO_4,_ etc. are added to ensure ionic strength in the polymerization mixture formulation. In the polymerization mixture of pyrrole and methylene blue, no additional salts are required because methylene blue itself acts as a supporting electrolyte [26]. Regarding the optimal pH values for polymerization of pyrrole and methylene blue, the sandwich-type of Ppy-PMB layer was formed. Such sandwich-type of the layer was obtained in two steps of polymerization of Ppy and PMB. In the first polymerization step, Ppy was electro-polymerized on the electrode from solution at low pH and in the second step, PMB was electro-polymerized from solution at high pH [27,28]. Such sandwich-type of Ppy-PMB layer was applied as a mediator in a microbial fuel cell with a Ppy-PMB composite electrode [27], and as an additive that was able to decrease significantly the charge transfer resistance of Ppy [28]. In previous studies, Ppy-PMB layers were also used for optoelectronic applications [26], as a photosensitizer for medical applications in deep tissue treatment [29], or in the solid-state photo-electrochemical cells [30]. Summarizing the aforementioned studies, we can see that although the Ppy and PMB co-deposited layer have been previously studied, the electrochromic properties of such a layer, especially of the Ppy-PMB doped with heparin, have not been studied yet. In addition, the possibility of applying the Ppy-PMB layer as an electrochromic sensor has been very little investigated. The most recent study of application of Ppy-PMB was published by our scientific group [31]. 

Therefore, the aim of this study was to co-deposit the Ppy-PMB layer on the ITO electrode in the presence of three saccharides (lactose, sucrose, and heparin) during a single-step procedure and compare the effect of different saccharides on the electrochromic properties. The properties of the layer were analyzed with respect to the changes of absorbance by changing the pH of the surrounding solution. The most mechanically stable layer was tested as an electrochromic sensor for xanthine derivatives. This study demonstrates that Ppy-PMB can be used in the design of electrochromic sensors.

## 2. Materials and Methods

### 2.1. Chemicals and Materials

Ultra-pure H_2_O was obtained by Crystal 7 water purification system received from Adrona (Riga, Latvia). The conductivity of purified water was 0.055 μS/cm. The pyrrole was purchased from Sigma-Aldrich (Darmstadt, Germany) and was distilled before use. Methylene blue from Alfa Aesar (Karlsruhe, Germany), lactose (Lac) from Carl Roth (Karlsruhe, Germany), sucrose (Suc) from Fluka (Darmstadt, Germany), heparin (Hep) from Rotexmedica (Trittau, Germany), and acetone from Reachem (Petržalka, Slovakia) were of analytical grade and were used as obtained. Ammonium hydroxide (27%) and hydrogen peroxide (30%) were from Chempur (Piekary Śląskie, Poland). Xanthine derivatives used in this study were theophylline, caffeine from Sigma Aldrich (Germany), and theobromine from Alfa Aesar (Germany). 

Britton–Robinson buffer (BRB) solution [33] was made of 0.01 M boric acid from Scharlau (Barcelona, Spain), 0.01 M acetic acid from Carl Roth (Germany), and 0.01 M phosphoric acid from Fluka (Germany). The ionic strength of BRB was supported with 0.1 M potassium chloride from Scharlau (Spain). The pH of solutions to the required value was adjusted with 1 M sodium hydroxide from Merck (Darmstadt, Germany).

### 2.2. Pretreatment of ITO-Coated Glass Slide 

Pretreatment of ITO-coated glass slide was carried out as it was described in a previous study [22]. ITO-coated glass slide (glass/ITO) with surface resistivity of 15–25 Ω/cm^2^ was purchased from Sigma-Aldrich (Steinheim, Germany). Glass/ITO surface was washed in a solution consisting of 27% NH_4_OH and 30% H_2_O_2_ mixed at ratio 3:1 and preheated up to 50 °C degrees for 4 min. Later on, glass/ITO was treated by ultrasound subsequently in water, acetone, and water for 15 min in each liquid. After the pretreatment procedure, the glass/ITO was dried out with argon stream and stored in dry conditions.

### 2.3. Co-Deposition of Ppy-PMB Layer on ITO Glass Electrode

A computer-controlled potentiostat/galvanostat PGSTAT 128N equipped with Nova 1.10 software from Eco-Chemie (Utrecht, The Netherlands) was used for electrochemical polymerization of conducting polymer and electrochemical measurements. A three-electrode system was applied for all electrochemical depositions. Ag/AgCl wire was used as a reference electrode and platinum wire as a counter electrode (CH Instruments, Austin, TX, USA). ITO electrode was used as a working electrode. The electrochemical deposition was carried out from a solution containing of 50 mM of pyrrole, 10 mM of methylene blue, and one of the doping materials: 0.1 M Lac, 0.1 M Suc, or 0.01 g/L Hep. The formed Ppy and PMB layers doped with saccharides were indicated as (Ppy-PMB)_Lac_, (Ppy-PMB)_Suc_, and (Ppy-PMB)_Hep_. The parameters of electrochemical polymerization were selected according to our previous research [31]. Polymerization was performed at a room temperature during 25 potential cycles in the range from −0.5 V to +1.2 V vs. Ag/AgCl, at the sweep rate of 50 mV/s and step potential of 2.44 mV.

### 2.4. Evaluation of Dominant Features by Atomic Force Microscopy and Scanning Electron Microscopy 

Visualization and evaluation of surface dominant features were performed by atomic force microscope (AFM) ‘Bioscope/Catalyst’ from Bruker (Santa Barbara, CA, USA). AFM images were obtained with a silicon nitride probe coated with a gold reflective layer (tip radius 20 nm, nominal resonant frequency 56 kHz, spring constant 0.24 N/m). Additionally, layers were evaluated using scanning electron microscope (SEM) TM4000-Plus from Hitachi (Hitachinaka, Japan).

### 2.5. Evaluation of Electrochromic Properties of Ppy-PMB Layer at Different PH of the Solution

A spectrometer USB4000-FL equipped with SpectraSuite software was purchased from Ocean Optics (Largo, FL, USA) and was used for optical measurements.

The changes of pH were followed with pH-meter ProLine Plus (Q-i-s, Oosterhout, The Netherlands) in BRB with 0.1 M KCl solution by increasing the pH with 1 M NaOH. Continuous measurements of the UV absorbance at particular wavelengths (668 nm and 750 nm) vs. time were performed at each pH value of BRB solution. Appropriate wavelengths were selected taking into account the absorbance spectra of methylene blue with the maximum at 668 nm [34] and of Ppy with the maximum at 750 nm [22].

Different reference electrodes were used during co-deposition of Ppy-PMB and evaluation of electrochromic response. Ag/AgCl_(3M KCl)_ (CH Instruments, Austin, TX, USA) was used as a reference electrode and platinum wire as a counter electrode. ITO electrode modified with Ppy-PMB layer was used as a working electrode.

A potential pulse sequence (PPS) was applied to evaluate the electrochromic properties of Ppy-PMB layer. Potential pulses were +0.8 V and −0.8 V. Duration of the pulse was 10 s. Each potential was repeated 5 times.

### 2.6. Application of (Ppy-PMB)_Hep_ Layer for Sensing Xanthine Derivatives

The ITO electrode coated with (Ppy-PMB)_Hep_ layer was used for sensing of xanthine derivatives. The changes of absorbance at different theophylline, theobromine, and caffeine concentrations and potential values were evaluated. The potential was alternated in BRB with 0.1 M KCl, pH 2.5 according to the following procedure: +0.8 V for 10 s, then −0.8 V for 10 s. Each potential was replicated 5 times. The changes of absorbance were registered at 668 nm and 750 nm wavelengths.

## 3. Results

In the previous study, it was mentioned that some destruction of Ppy-Phenothiazine (Ppy-PT) layer on ITO electrode was observed at a certain pH value [31]. To overcome such limitation, some measures can be applied: (1) modification of the ITO electrode surface with silanes without specific functional groups in order to form the silane-based self-assembled monolayers (SAM) and so to improve the non-covalent interaction of the polymer layer with electrode surface [22] or with silane-based SAM with pyrrolyl end-groups, e.g., 11-(1H-Pyrrol-1-yl)undecane-1-thiol; (2) doping with some polysaccharides, for example, heparin, which can be electrochemically incorporated into Ppy to enhance adhesion [19,24].

**Figure 2:**
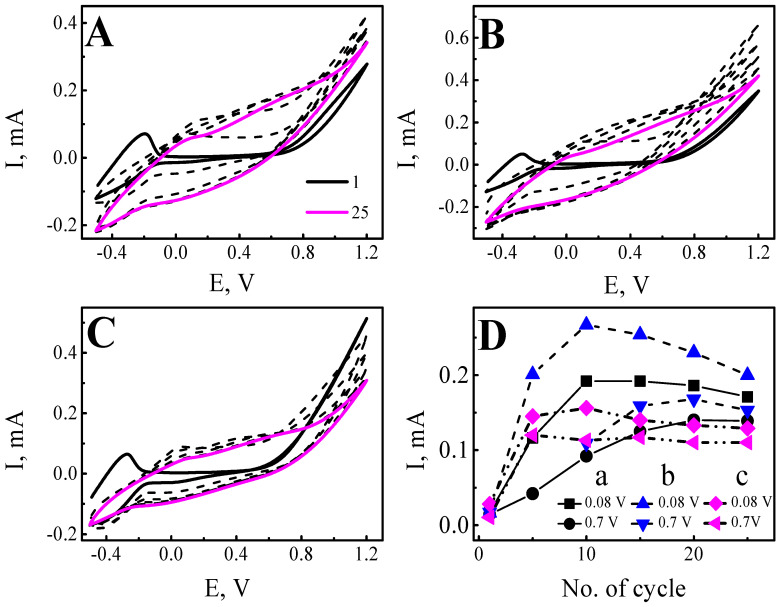
Electropolymerization of Ppy-PMB layers by potential cycling. Voltammograms at different cycles (1, 5, 10, 15, 20, 25): (**A**)—(Ppy-PMB)_Lac_, (**B**)—(Ppy-PMB)_Suc_, (**C**)—(Ppy-PMB)_Hep_, (**D**)—the width of the cycle loop in mA (Δ*I*) was compared at potentials +0.08 V and +0.7 V. a—(Ppy-PMB)_Lac_, b—(Ppy-PMB)_Suc_, c—(Ppy-PMB)_Hep_. Polymerization solution consisted of 50 mM pyrrole, 10 mM methylene blue, and 0.1 M lactose, 0.1 M sucrose, or 0.01 g/L heparin. Ppy-PMB layers were obtained by potential cycling 25 cycles, from −0.5 V to +1.2 V, at scan rate of 50 mV/s, and step potential of 2.44 mV. WE—glass/ITO, RE—Ag/AgCl wire, CE—platinum wire.

Electrochemical polymerization of Ppy-PMB layers doped with lactose (Lac), sucrose (Suc), and heparin (Hep) was carried out by conditions described in Section 2.3. In total, 25 potential cycles were applied to obtain the layers. The voltammograms obtained during electropolymerization by potential cycling are represented in Figure 2A–C. Comparison of the current changes obtained during the first cycle demonstrates the visible cathodic peak of current at the beginning of the oxidation process at −0.20 V for the (Ppy-PMB)_Lac_, −0.28 V for the (Ppy-PMB)_Suc_, and −0.277 V for the (Ppy-PMB)_Hep_ layers. This increment of current could identify the beginning of polymer formation on the ITO electrode. During the next potential cycles, it was observed that the cathodic peak was moved to the higher potential values.

The comparison of the current changes obtained during electropolymerization of Ppy-PMB described in this and other studies demonstrate that the shift of cathodic peak depends on the type of electrode and other polymerization conditions, such as composition of the solution, pH, etc. The cathodic peak of methylene blue on platinum foil was at −0.22 V and it is attributed to the oxidation of methylene blue [14]. The oxidation peak of methylene blue during electropolymerization on the gold electrode moved to more negative potential values (≈−0.4 V) [15], whereas the oxidation peak (≈−0.255 V) of methylene blue during electro-polymerization on glassy carbon electrode was very similar as it was obtained in this study on ITO electrode. Thus, this confirms that the cathodic peak at the ≈−0.2 V (Figure 2A–C) is attributed to the oxidation of methylene blue.

The oxidation peak of pyrrole usually is less expressed than the oxidation peak of methylene blue. In the previous study, it was demonstrated that polymerization of pyrrole on ITO electrode depending on the modification of electrode surface started at +0.53 and +0.70 V vs. Ag/AgCl_(3M KCl)_ [22]. It is notable that the oxidation potentials get lower with increasing chain length [35]. In this study, the nucleation of pyrrole during electro-polymerization of Ppy-PMB (Figure 2A–C) is not clearly expressed and cannot be clearly distinguished from non-Faradaic processes. 

Evaluation of the width of the cycle loop Δ*I* (Figure 2D) gives a deeper insight into electrical conductivity changes of the Ppy-PMB layer during the electropolymerization process. The width of the cycle loop was compared at two potential values: +0.08 V and +0.7 V. An increase in loop width identifies the growth of the polymer layer. However, approximately from the 10th cycle, a decrease in the loop width can be attributed to some overoxidation of the Ppy layer as it was demonstrated in the previous study [21]. Moreover, it was stated that cause of the induced overoxidation process could be nucleophilic solvents, for instance, water [35].

**Figure 3:**
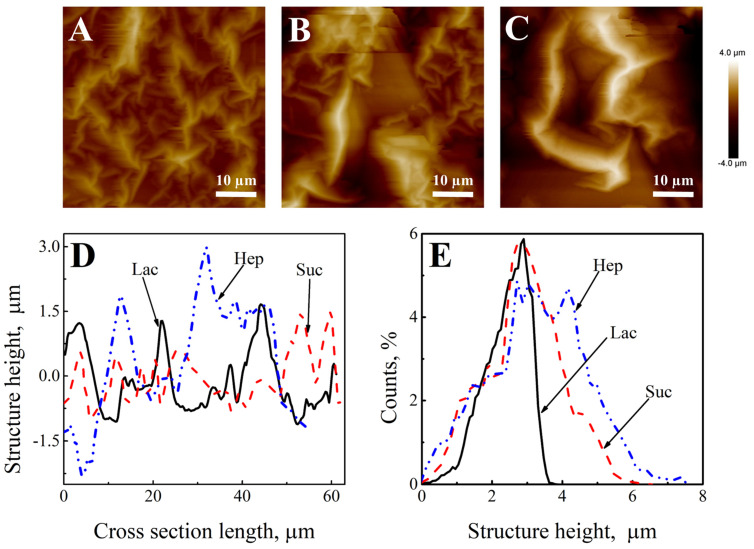
The AFM-based evaluation of surface morphology dominant features of (**A**)—(Ppy-PMB)_Lac_; (**B**)—(Ppy-PMB)_Suc_; (**C**)—(Ppy-PMB)_Hep_. (**D**)—The comparison of the cross-sections of surfaces of the layers. (**E**)—Height distribution. Electrochemical deposition conditions are indicated in Figure 1.

Surface morphology and roughness were analyzed by AFM (Figure 3). The dominant features of all three electrodes surfaces were fairly different. Figure 2B,C and Figure 3A demonstrate the appearance of the layers. It can be seen that the surface of the (Ppy-PMB)_Lac_ (Figure 3A) layer is most evenly distributed. The most massive surface structures were formed on the (Ppy-PMB)_Hep_ layer (Figure 3C). From the length of cross-section vs. structure height distribution (Figure 3D), it is obvious that the height is up to 1.5–2.5 μm for (Ppy-PMB)_Lac_, up to 2 μm for (Ppy-PMB)_Suc_, and up to 3 μm for (Ppy-PMB)_Hep_. Figure 3E depicts surface height distribution and confirms that dominant height is around 2.5 μm for all Ppy-PMB layers. It confirms the previous observations about layers’ surface roughness. From the AFM images, it could be seen that all layers have wrinkles on the surface from smaller ((Ppy-PMB)_Lac_) to larger ((Ppy-PMB)_Hep_). These wrinkles could be formed during the drying process of the layer after synthesis procedure. Possibly, in a solution, the synthesized layer is filled up with water molecules that evaporate during drying procedure. Different wrinkle size could be due to a different Ppy-PMB layer thickness or the strength and compactness of the structure itself. Therefore, from the analysis by AFM it can be concluded that the saccharides used in this study have a moderate effect on the surface morphology and roughness of Ppy-PMB layers.

**Figure 4:**
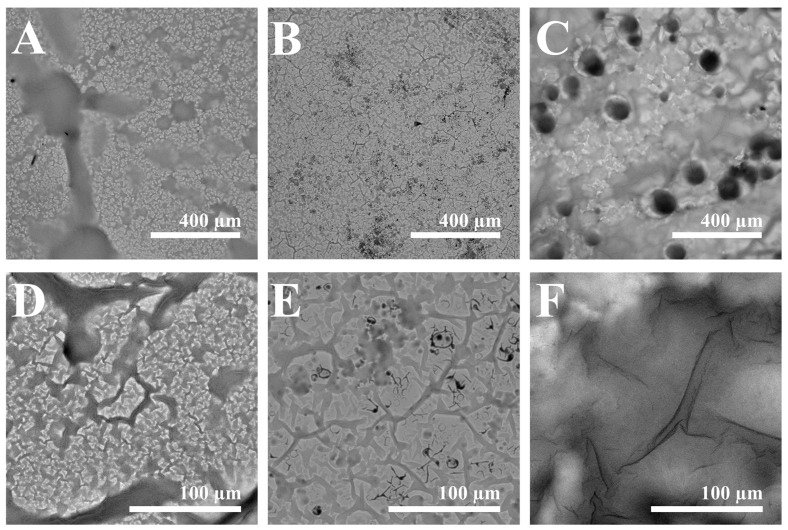
SEM images of (**A**,**D**)—(Ppy-PMB)_Lac_, (**B**,**E**)—(Ppy-PMB)_Suc_, (**C**,**F**)—(Ppy-PMB)_Hep_. Magnification for (**A**–**C**) was × 200 and for (**D**–**F**) was × 1000. Imaged area for (**A**–**C**) is of 1125 μm × 1125 μm and for (**D**–**F**) is 225 μm × 225 μm.

The structure of the obtained layers was characterized using SEM-based imaging. These images also confirmed the AFM analysis. The surface of (Ppy-PMB)_Lac_ is represented in Figure 4A,D and is evenly distributed, some polymer agglomerations are formed. Figure 4B,E represent surface images of (Ppy-PMB)_Suc_ layer, which is also fairly evenly distributed, the structures are minor, but the folds are more expressive than in the previous layer. In Figure 4C,F, the surface of (Ppy-PMB)_Hep_ layer is illustrated. The surface structure of this layer is the most massive and wrinkles are the largest. Thus, SEM results verify AFM images and conclusions, that the largest wrinkles are formed using heparin and the smallest in the sample with lactose.

**Figure 5:**
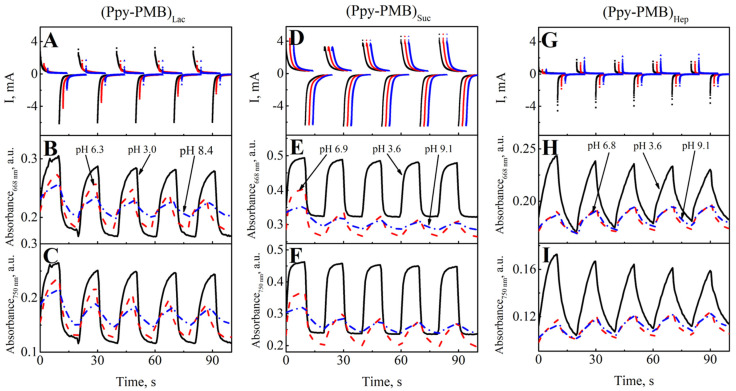
Evaluation of Ppy-PMB layers during potential pulsation. (**A**)—potential pulse sequence of (Ppy-PMB)_Lac_, (**D**)—(Ppy-PMB)_Suc_, (**G**)—(Ppy-PMB)_Hep_ (pH 6 offset 2, pH 9 offset 4). Changes of absorbance at different pH values (at pH 3.0–4.0, pH 6.0–7.0, and pH 8.0–9.0): for (Ppy-PMB)_Lac_ at (**B**)—λ = 668 nm, and (**C**)—λ = 750 nm; for (Ppy-PMB)_Suc_ at (**E**)—λ = 668 nm, and (**F**)—λ = 750 nm; and for (Ppy-PMB)_Hep_ at (**H**)—λ = 668 nm and (**I**)—λ = 750 nm. Potential pulses were +0.8 V and −0.8 V. Duration of the pulse was 10 s. Each potential was repeated 5 times. The dependence of optical absorbance was measured in BRB with 0.1 M of KCl solution. WE—glass/ITO electrode with Ppy-PMB layer, CE—platinum wire, RE—Ag/AgCl_(3M KCl)_.

In the further part of the study, the layers were examined by varying the pH of the BRB solution and the potential. The pH of the BRB solution was increased using 1 M NaOH from pH 2.5 up to pH 9. The sequence of potential pulses was applied to evaluate the absorbance changes with respect to the changing potential of Ppy-PMB layers at each pH value. Potential pulses were +0.8 V and −0.8 V. Duration of the pulse was 10 s. Each potential pulse was repeated 5 times. The pH value of BRB solutions was stepwise increased from acidic to basic and the absorbance at the wavelengths 668 nm and 750 nm was followed (Figure 5). The tendency to increase the absorption at the positive potentials and to decrease at the negative potentials was observed.

In previous studies, the evaluation of absorbance spectra of PMB when potential was changed from −0.3 V to +0.5 V by an increment of potential by 0.1 V demonstrated that the position of absorbance peaks at 613 nm and 654 nm do not change with the potential values [14]. The main significant changes occur in the ratio of absorbance at these two wavelengths and which are assigned to the absorption bands of the monomeric and dimeric forms of methylene blue [26]. Another study reported that Ppy doped with Hep is an effective strategy to enhance the electrochromic lifetime of polymer films [24]. The investigated layer was characterized by long-term electro-optical stability: the optical contrast changed from 48% to 42% after 100 steps. In the case of our study, the resulting (Ppy-PMB)_Hep_ layer was incomparably more mechanically stable on the electrode than (Ppy-PMB)_Lac_ and (Ppy-PMB)_Suc_ layers. However, response times were more than two times longer than they were in the recent study and the absorption curves are not as orderly as the other two layers studied. It can be assumed that the same electro-optical stability cannot be seen due to the incorporation of methylene blue.

During the comparison of absorbance at the wavelength 668 nm (Figure 5B,E,H) and 750 nm (Figure 5C,F,I), it was observed that the value of optical absorbance for the (Ppy-PMB)_Suc_ layer is greater than that for the (Ppy-PMB)_Lac_ and (Ppy-PMB)_Hep_. The intensity of optical absorbance decreases when the medium becomes more alkaline (Figure 5).

Analysis of the (Ppy-PMB)_Lac_ layer showed that in all cases, the absorption maximum decreases with the increasing number of pulses, for example, in an acidic medium the absorption ranges from 0.30 to 0.280 at 668 nm and from 0.264 to 0.242 at 750 nm. At neutral and basic pH values, the layer becomes less stable to potential change, resulting in a reduction in the difference between the absorption maxima. As the medium becomes more basic, a drop in values is seen, but it is not as abrupt as when observing other layers. In the case of the (Ppy-PMB)_Suc_ layer, the absorption values at 668 nm wavelengths range from 0.494 to 0.478 and at 750 nm wavelengths from 0.462 to 0.450. At a more alkaline pH, it can be seen that (Ppy-PMB)_Suc_ at both wavelengths reaches the highest absorption maximum during the first potential pulse and decreases markedly during the second pulse. From the second pulse, the absorption narrows slightly at these two pH values. The electrochromic properties of the (Ppy-PMB)_Suc_ layer are best expressed in acidic pH. The (Ppy-PMB)_Hep_ layer exhibits rather similar properties and tendencies in an acidic medium as the other two, but in the presence of neutral and acidic media, a slight increase in the absorption maximum is seen. 

Δ*A* was calculated by using the following Equation (1):

(1) $$\Delta A=A_{+0.8V}-A_{-0.8V}$$

**Figure 6:**
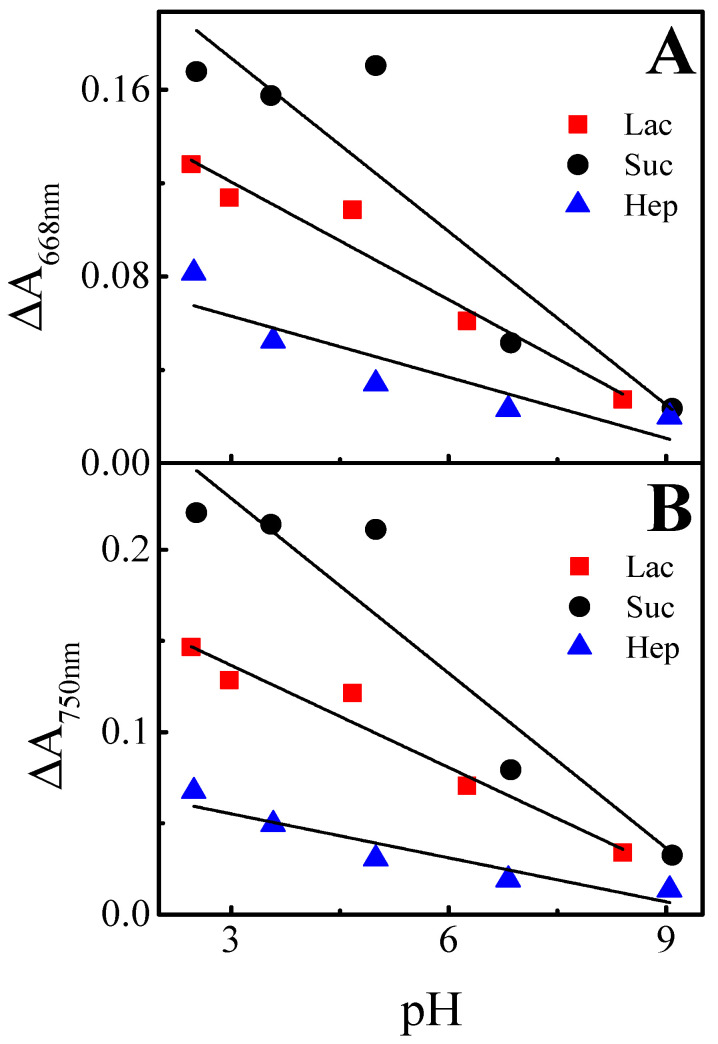
Calibration curve of Δ*A* (at the wavelengths of: (**A**)—λ = 668 nm and (**B**)—λ = 750 nm) as function of the pH value of BRB with 0.1 M of KCl solution, when potential pulses were of +0.8 V and –0.8 V with duration of 10 s. Each potential pulse was repeated for 5 times.

For the calculation of Δ*A*, the values of *A*_+0.8*V*_ and *A*_−0.8*V*_ at the end of the potential pulse were taken. It was found that Δ*A* was the highest in the most acidic solutions regardless of the wavelength and it decreased for all Ppy-PMB layers when the pH of the solution was changed to more alkaline (Figure 6 and Table 1).

Thus, we can conclude that by changing the pH of the solution and potential value from +0.8 V to −0.8 V, the decrease of the change of absorbance can be observed. Therefore, the highest absorbance changes are observed in the acidic pH of the solution for all Ppy-PMB layers. A similar conclusion was drawn and for the Ppy layers but without PMB [22].

Studies have shown that combining conductive polymers with tunable coloration can lead to smarter electrochromic devices [1]. Electrochromism can be characterized by optical contrast, which is the transmittance difference between the bleached and colored states.

The electrochromic property of optical contrast was also analyzed. Response times were very similar in all cases. Changing the pH to the alkaline side showed visible change—slowing down response times in coloration and bleaching. In the case of (Ppy-PMB)_Lac_ layers at 668 nm, a response time of about 8 s for coloration and 2.8 s for bleaching was shown. In the case of (Ppy-PMB)_Suc_, there was a response time of about 5.9 s for coloration and 2.1 s for bleaching. In the case of (Ppy-PMB)_Hep_, response times were the longest compared to others: about 9.8 s for coloration and 3.6 s for bleaching. Very similar results were obtained at 750 nm. 

The last part of the study was attributed to the application of the formed Ppy-PMB layer for sensing three xanthine derivatives: theophylline, caffeine, and theobromine. Such xanthine derivatives were selected taking into account that these compounds: (1) are soluble in water-based solutions (solubility in water are 0.330 g/L of theobromine, 7.360 g/L of theophylline, and 21.6 g/L caffeine of at 25 °C); (2) xanthine derivatives differ from each other only by a methylene group, therefore they are convenient for model systems. Xanthine derivatives are found in the biological fluids of humans and some other species and are receiving a lot of interest because of their bio-activity. They are used as pharmaceutics because they have such bio-activities: antimicrobial, antiasthmatic, CNS stimulating, anti-inflammatory, and antagonism to adenosine receptor [8,36,37]. Methylxanthine homologues include such compounds such as caffeine, theophylline, and theobromine. Methylxanthines have some typical parameters suitable for analytical applications. 

In previous studies, some applications of polyphenazines for the determination of xanthine derivatives were described [11,38,39]. The study by Bukkitgar and Shetti [39] described the electrochemical behavior of theophylline at the methylene blue dye modified carbon paste. The main analytical methods used for the determination of theophylline were cyclic voltammetry and differential pulse voltammetry (DPV). The obtained results demonstrated that modification of carbon paste electrode with methylene blue during analysis by cyclic voltammetry CV leads to the peak current of theophylline sharpening and increased current values about 2–3 folds in comparison to the peak current obtained on the bare carbon paste electrode. Ppy was also used for the determination of theophylline [4,40,41,42]. The mentioned studies applied molecular imprinting technology for improved sensitivity/selectivity of theophylline on Ppy by electrochemical analysis (cyclic voltammetry, electrochemical impedance spectroscopy (EIS)) or microgravimetric (quartz crystal microbalances (QCM)) methods.

In comparison to the previously published studies, the experiment described in this article is unique. First of all, this time the combination of polypyrrole and methylene blue was applied as a sensor of xanthine derivatives. In contrast to the previously published study where carbon paste electrode modified with PMB was used as a working electrode [39], in this study, ITO modified with (Ppy-PMB)_Hep_ was used as a working electrode. Secondly, in this study, the (Ppy-PMB)_Hep_ layer was applied as a possible electrochromic surface for the xanthine derivatives detection.

**Figure 7:**
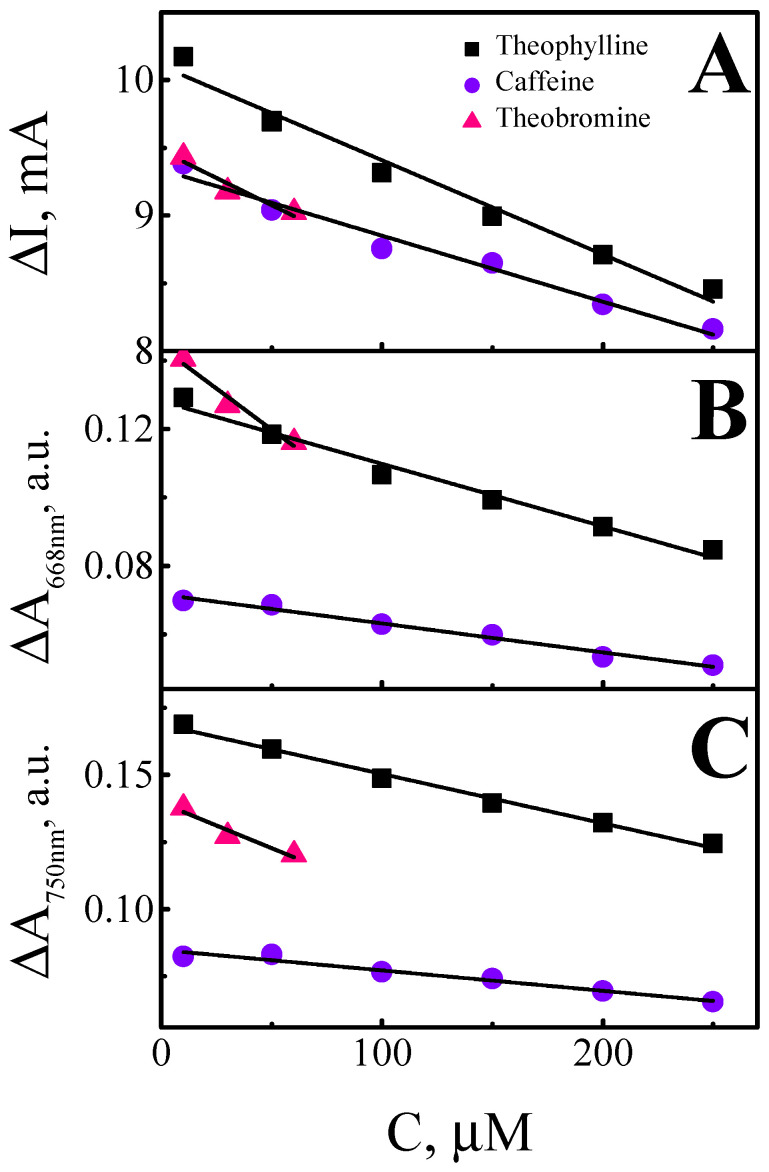
Dependence of absorbance changes (at λ = 668 nm and λ = 750 nm) on the concentration of caffeine, theophylline, and theobromine in BRB with 0.1 M of KCl, pH 2.5 solution. (**A**)—calibration curves of Δ*I* vs. concentration of caffeine, theophylline, and theobromine, (**B**)—calibration curves of Δ*A* (at 668 nm) vs. concentration of caffeine, theophylline, and theobromine, (**C**)—calibration curves of Δ*A* (at 750 nm) vs. concentration of caffeine, theophylline, and theobromine.

The heparin-doped (Ppy-PMB)_Hep_ layer was chosen to sense xanthine derivatives as the most mechanically stable layer. Evaluation of absorbance dependence (at λ = 668 nm (Figure 7B) and λ = 750 nm (Figure 7C)) on the concentration of caffeine, theophylline, and theobromine was performed in BRB solution with 0.1 M of KCl, pH 2.5. The concentration interval of the theobromine was limited by the compound solubility in the BRB solution. The pH value of BRB solution was selected based on results in the prior section where it was demonstrated that the absorbance changes in the most acidic BRB solution was highest. The potential pulses profile was used unchanged: +0.8 V and −0.8 V with a duration of 10 s. 

It was obtained that absorbance dependence (at λ = 668 nm and λ = 750 nm) on the concentration of caffeine, theophylline, and theobromine was linear and the correlation coefficient R^2^ for the Δ*A* at wavelength 668 nm and 750 nm was even higher than for the Δ*I* (Figure 7A). 

Linearity was observed at all evaluated calibration curves of xanthine derivatives. The slope was derived using the linear regression equation for the changes of absorbance changes (at λ = 668 nm and λ = 750 nm) vs. the concentration of xanthine derivatives (c, μM). In the case of caffeine (at λ = 668 nm) slope of linear regression was −0.001 with R^2^ = 0.978 (Table 2), while in the case of theophylline slope was −0.002 with R^2^ = 0.977 (Table 2), which is 2 times higher. In the case of theobromine, the slope of linear regression was −0.005 with R^2^ = 0.939. A linear relationship is observed only in low concentrations, followed by scattering of points, so only the first three points are reported. The limit of detection (LOD) as the lowest concentration of analyte, which gives an analytical signal greater than the background value plus 3 δ, was estimated. The LODs for Δ*I* vs. concentration of caffeine, theophylline, and theobromine were 2.0 μM, 3.9 μM, and 7.48 μM, respectively. The LODs for caffeine, theophylline, and theobromine based on registered changes of absorbance at λ = 668 nm were 3.3 μM, 3.0 μM, and 6.0 μM, respectively; while changes of absorbance registered at λ = 750 nm LODs were 3.75 μM, 3.0 μM, and 3.0 μM, respectively. Accordingly to the given results, it is presupposed that the (Ppy-PMB)_Hep_ layer might be used for the detection of xanthine derivatives, especially for theophylline and caffeine.

## 4. Conclusions

The electrochemical deposition of the polypyrrole layer was carried out by cyclic voltammetry from a solution containing of 50 mM of pyrrole, 10 mM of methylene blue, and one of the doping materials: 0.1 M Lac, 0.1 M Suc, 0.01 g/L Hep. Surface morphology was analyzed by AFM and SEM methods. From the AFM analysis, these layers were compared with each other. It can be concluded that the saccharides used in this study have a moderate effect on the surface morphology and roughness of Ppy-PMB layers. From the length of cross-section vs. structure height distribution, it is obvious that the height is up to 1.5–2.5 μm for (Ppy-PMB)_Lac_, up to 2 μm for (Ppy-PMB)_Suc_, and up to 3 μm for (Ppy-PMB)_Hep_.

The Ppy-PMB layers were examined by varying the pH of the BRB solution and the potential. The tendency to increase the absorption at the positive potentials and to decrease at the negative potentials was observed at each pH value. It was observed that the value of optical absorbance for the (Ppy-PMB)_Suc_ layer is greater than that for the (Ppy-PMB)_Lac_ and (Ppy-PMB)_Hep_. Moreover, it was found that the intensity of optical absorbance itself and the change of absorbance was the highest in the most acidic solutions regardless of the wavelength and it decreased for all Ppy-PMB layers when the pH of the solution was changed to more alkaline. The (Ppy-PMB)_Suc_ layer had the best conductivity properties, and the fastest bleaching and coloration times. The suitability of the Ppy-PMB layer for use as an electrochromic sensor of xanthine derivatives—caffeine, theophylline, and theobromine—was investigated using the (Ppy-PMB)_Hep_ layer. Linearity was observed at evaluated calibration curves (concentration vs. the change of absorbance) of all xanthine derivatives. According to the given results, it is presupposed that the (Ppy-PMB)_Hep_ layer might be used for the detection of xanthine derivatives, especially for theophylline and caffeine.

## Figures and Tables

**Table 1:** Dependence of absorbance changes (at λ = 668 nm and λ = 750 nm) on the pH of BRB solution. The slope and intercept values of the calibration curves.

Wavelength	Layer	Slope	Intercept	R^2^
668 nm	(Ppy-PMB)_Lac_	−0.017 ± 0.002	0.171 ± 0.001	0.935
	(Ppy-PMB)_Suc_	−0.025 ± 0.006	0.248 ± 0.036	0.790
	(Ppy-PMB)_Hep_	−0.009 ± 0.002	0.089 ± 0.0145	0.742
750 nm	(Ppy-PMB)_Lac_	−0.019 ± 0.002	0.193 ± 0.012	0.944
	(Ppy-PMB)_Suc_	−0.032 ± 0.006	0.324 ± 0.038	0.857
	(Ppy-PMB)_Hep_	−0.008 ± 0.002	0.079 ± 0.010	0.851

It was observed that the Δ*A* value of the (Ppy-PMB)_Suc_ layer dropped by 86% at 750 nm and 668 nm wavelengths. The Δ*A* value of (Ppy-PMB)_Lac_ and (Ppy-PMB)_Hep_ layers changed in a descending direction, too. The Δ*A* value of the (Ppy-PMB)_Lac_ layer dropped by 80% at 750 nm and 78% at 668 nm. In the case of the (Ppy-PMB)_Hep_ layer, the Δ*A* value dropped by 78% at 750 nm and 81% at 668 nm.

**Table 2:** Dependence of absorbance changes (at λ = 668 nm and λ = 750 nm) on the concentration of caffeine, theophylline, and theobromine. The slope and intercept values of the calibration curves.

		Slope	Intercept	R^2^
Δ*I*	Theophylline	−0.007 ± 0.0005	10.1 ± 0.079	0.973
	Caffeine	−0.005 ± 0.0004	9.35 ± 0.059	0.969
	Theobromine	−0.008 ± 0.0021	9.48 ± 0.082	0.872
668 nm	Theophylline	−0.0002 ± 0.00001	0.13 ± 0.002	0.977
	Caffeine	−0.0001 ± 0.00001	0.07 ± 0.001	0.978
	Theobromine	−0.0005 ± 0.00009	0.14 ± 0.003	0.939
750 nm	Theophylline	−0.0002 ± 0.00001	0.17 ± 0.001	0.990
	Caffeine	−0.0001 ± 0.00001	0.08 ± 0.001	0.958
	Theobromine	−0.0003 ± 0.00008	0.14 ± 0.003	0.884

## Data Availability

The data presented in this study are available on request from the first author.
